# Two-stage exchange Arthroplasty for knee Periprosthetic joint infection exhibit high infection recurrence rate in patients with chronic viral hepatitis

**DOI:** 10.1186/s12891-021-04416-0

**Published:** 2021-06-12

**Authors:** Jui-ping Chen, Chih-hsiang Chang, Yu-chih Lin, Sheng-hsun Lee, Hsin-nung Shih, Yuhan Chang

**Affiliations:** 1 Department of Orthopaedic Surgery, Chang Gung Memorial Hospital, Linkou, Taiwan; 2 College of Medicine, Chang Gung University, Taoyuan, Taiwan; 3 Bone and Joint Research Center, Chang Gung Memorial Hospital, Linkou, Taiwan; 4 Department of Orthopaedic Surgery, Chang Gung Memorial Hospital, 5, Fu-Hsin St., Kweishan, Taoyuan, Taiwan

## Abstract

**Background:**

Currently, there is little evidence about the outcome of two-stage exchange arthroplasty for the treatment of knee periprosthetic joint infection (PJI) in patients with chronic viral hepatitis. To evaluate it, we set the primary outcome as infection recurrence, and the secondary outcome as the difference between patients diagnosed with hepatitis B virus or hepatitis C virus.

**Patients and methods:**

Between June, 2010 and December, 2016, 172 patients with knee PJIs were treated with two-stage exchange arthroplasty at our institute. Treatment success was defined using Delphi-based consensus. These patients were further divided into groups with or without chronic hepatitis. Variables were analyzed, including age, sex, comorbidities, microbiology, and operative methods. Minimum follow-up was 12 months (mean, 35 months; range, 12-85 months).

**Results:**

Of the 172 knee PJI patients, 25 were identified with chronic hepatitis. The infection recurrence rate in the hepatitis group (28%, 7 in 25) was significantly higher than that in the non-hepatitis group (9.5%, 14 in 147), *p* = 0.017. However, there was no significant difference in the infection recurrence rates between patients with HBV (24%, 4 in 16) and HCV (33.3%, 3 in 9). Regarding the outcomes of patients with infection recurrence, 4 of the non-hepatitis patients were treated with the debridement, antibiotic treatment, irrigation, and retention of prosthesis (DAIR) procedure, with a success rate of 75%. The other 17 patients (7 with hepatitis and 10 without) were treated with repeated two-stage exchange arthroplasty with 100% infection elimination rate until the final follow-up.

**Conclusions:**

Knee PJI patients with chronic hepatitis have higher infection recurrence rate after two-stage exchange arthroplasty (28%).

## Introduction

Over the past decade, there has been an increase in the number of knee arthroplasties performed worldwide. Over a 10-year period the incidence of knee periprosthetic joint infection (PJI) is about 0.75% at 10 years in primary TKA, accounting for nearly one-fourth in revision cases [1, 2]. Post-operative complication rate of revision TKA for PJI is high (13.7%) [3], and 5-year mortality rate is 25.9% [2].

Patients can be more susceptible to PJI with specific risk factors, such as uncontrolled diabetes [4–6], malnutrition [7], obesity [8], smoking [9], and chronic hepatitis. Chronic hepatitis can induce immune dysfunction and systemic inflammation, causing high incidence of knee PJI. Recent studies have reported high complication rates after hip and knee arthroplasty in patients with chronic hepatitis [10–12].

In early acute PJI, debridement, antibiotic, and implant retention (DAIR) procedure showed satisfying treatment result [13]. Two-stage exchange arthroplasty has been generally used as gold-standard to treat chronic PJI, and the success rate is between 64 and 89.9% [14–17]. Several risk factors have been reported to be associated with failure of the treatment for knee PJI, including obesity, smoking, heart disease, psychiatric disorders, female gender, and polymicrobial infection [18–20]. In immunocompetent patients whose infecting micro-organism and sensitivity are verified, one-stage exchange arthroplasty can be a valuable choice of treatment [21].

To the best of our knowledge, the efficacy of two-stage exchange arthroplasty in knee PJI patients with chronic hepatitis has not been reported. Chronic viral hepatis has been considered to have poor immune function and a high risk of infection [22]. This study was determined to evaluate the efficacy of two-stage exchange arthroplasty for the treatment of knee PJI. Our hypothesis is that patients with chronic hepatitis will have poorer outcomes after two-stage exchange arthroplasty for knee PJI.

## Methods

### Patient enrollment

We retrospectively reviewed a joint database of arthroplasty to identify patients who were diagnosed with knee PJI and were treated with two-stage exchange arthroplasty at our institution between June, 2010 and December, 2016. Patients who previously received revision surgery in other institutions were excluded.

PJI was defined by fulfilling one of the following three criteria: (1) a sinus tract communicating with the prosthesis; (2) isolated pathogens from two or more samples obtained from the infected prosthetic joint; (3) presence of purulence in the affected joint with elevated synovial white blood cell count and synovial neutrophil percentage (PMN%) combined with serum erythrocyte sedimentation rate and serum C-reactive protein concentration elevation [23].

Eight surgeons were involved in these operations, all following the same protocol. Pre-operative joint aspiration for microbiology culture was performed in all the enrolled patients. After diagnosis of knee PJI, they were treated with protocol of two-stage exchange arthroplasty. In brief, medial para-patella approach, resection arthroplasty for PJI included radical debridement, removal of prosthesis, antibiotic-loaded bone cement implantation, and administration of systemic antimicrobial agents for controlling joint infection. Vancomycin and Ceftazidime were loaded in Simplex P, Stryker, providing broad-spectrum anti-bacterial capacity [24]. All patients were under general anesthesia and torniquet was used in the whole course of operation. Intraoperative samples were sent for microbiology culture. Most of the wound can be closed without difficulty. In some case with potential soft tissue problem, negative pressure tissue therapy system was applied. Delayed re-implantation of the prosthesis after successful antimicrobial therapy was defined as no signs of infection and erythrocyte sedimentation rate and normal levels of serum C-reactive protein [25]. The choice of implant, whether primary or revision system, was based on patients’ bone loss and ligament condition.

Two to 4 weeks of antibiotics were used after the implant removal (first stage). Partial weightbearing was recommended in this interim period. One to 3 days of antibiotics were used after re-implantation of prosthesis (second stage). Walking with full weightbearing was allowed immediately after the re-implantation of prosthesis. The choice of regiment was depended on the microbiology culture. The outcome was assessed according to the 2013 Delphi-based International Consensus definition (1) infection eradication, characterized by a healed wound without fistula, drainage, or pain, and no infection recurrence caused by the same organism strain; (2) no subsequent surgical intervention for infection after reimplantation surgery; and (3) no PJI-related mortality caused by sepsis or necrotizing fasciitis.

We also identified patients who were diagnosed with chronic viral hepatitis, including hepatitis B virus (HBV) and hepatitis C virus (HCV). Patients were further divided into the hepatitis group and the non-hepatitis group (control group). Patients with liver cirrhosis were further identified within these cases, and liver cirrhosis was mainly diagnosed by histological findings. For those who had not undergone liver biopsy, the diagnosis of liver cirrhosis was made by at least two of the followings: (1) abnormal liver functional test (serum albumin ≤3.4 g/dl, international normalized ration [INR] ≤ 1.3, or serum bilirubin ≤2 mg/dl); (2) esophageal varices (EVs) on endoscope, and (3) portal hypertension suggested on imaging studies (ultrasonography or computed tomography) [26]. Hepatic function reservation was evaluated by the Child-Turcotte-Pugh (CTP) scoring system [27].

We reviewed patient characteristics (age, sex, BMI), comorbidities, results of microbiologic studies, and final outcomes in all knee PJI patients with or without chronic hepatitis. Approval for this study was obtained from the Institutional Review Board.

### Statistical analysis

The survival analysis was evaluated using Kaplan-Meier survival curve with 95% confidence interval (CI). The Chi-square test or the Fisher’s exact test was used where appropriate to analyze categorical data. Independent t-test or nonparametric Mann-Whitney U-test was applied for between-group comparisons in numerical data. Kaplan-Meier and Cox regression were applied for survival analysis. Statistical significance was defined as *p* < 0.05. Due to the small sample size in this study, univariate and two-sided analysis were performed in this study. Statistical analysis was carried out by SPSS 24.0 program for Windows (IBM SPSS Statistics for Windows, Version 24.0; IBM Corp, Armonk, NY, USA).

## Results

Between June, 2010 and December, 2016, 172 knee PJI patients (81 male and 91 female) with an average age of 68 years old (range, 25–87 years) were treated with two-stage exchange arthroplasty protocol at our institute. The mean duration of follow-up was 35 months (range, 12-85 months). Twenty-five patients were diagnosed with chronic viral hepatitis (16 HBV and 9 HCV), and 147 patients were hepatitis-free. The patients in both groups were homogeneous.

**Fig. 1:**
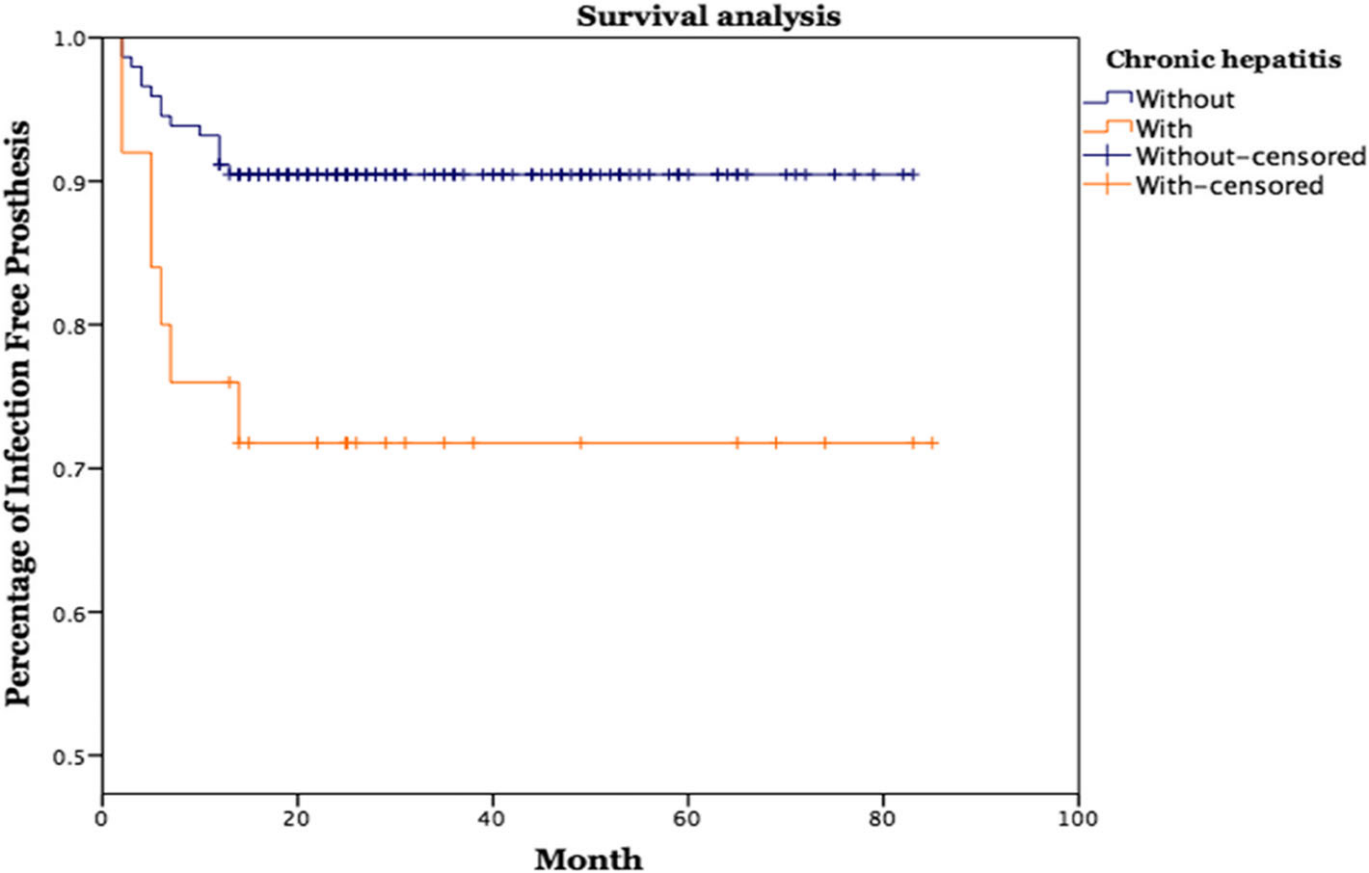
Kaplan-Meier Survival analysis of knee periprosthetic joint infection treated with two-stage exchange arthroplasty in patient with or without chronic viral hepatitis

The infection recurrence rate in the hepatitis group (28%, 7 in 25) was significantly higher than that in the control group (9.5%, 14 in 147), *p* = 0.017. Kaplan-Meier and Cox regression survival analysis showed a significantly higher treatment failure rate in the hepatitis group than in the non-hepatitis group, *p* = 0.01 (Fig. 1). There was no significant difference between HBV (24%, 4 in 16) and HCV groups (33.3%, 3 in 9). According to the Delphi-based consensus definition, the average infection recurrence time was 7 months (range, 2-14 months) after two-stage exchange arthroplasty. The Charlson Comorbidity Index (CCI) score was significantly higher in the hepatitis group than in the non-hepatitis group (5 vs. 4, *p* = 0.011). Details of the patient characteristics are summarized in Table 1.

**Table 1:** Demographics and characteristics of patients with or without chronic hepatitis

Number	Non-hepatitis	Hepatitis	*P*
	147	25	
Age	68 (25-87)	63 (30-83)	0.075
Sex			0.922
Male	69 (46.9%)	12 (48%)	
Female	78 (53.1%)	13 (52%)	
Follow up months	34 (12-83)	39 (13-85)	0.286
Infection relapse PJI	14 (9.5%)	7 (28%)	0.017**
Charlson comorbidity index	4 (0-11)	5 (1-7)	0.011**
Treatment after failure			
Second revision	10	7	
DAIR, success	3	0	
DAIR, fail, 2nd revision	1	0	

** *P* < 0.05

Regarding the 21 patients with infection recurrence, 4 patients (all in the non-hepatitis group) with infection signs/symptoms less than 3 weeks were treated with the DAIR procedure. One of them experienced treatment failure and took a second two stage exchange arthroplasty. The overall success rate of the first time DAIR in the non-hepatitis group was 75%. The other 17 patients (10 in the non-hepatitis group and 7 in the hepatitis group) with infection relapse sign/symptoms more than 3 weeks received repeated two-stage exchange arthroplasty, with 100% success rate.

Regarding the microorganisms in the knee PJI, *Staphylococcus aureus* was the most common pathogen (49 cases, 28.5%), followed by coagulase negative Staphylococcus (32 cases, 18.6%), *Enterococcus faecalis* (6 cases, 3.4%), and *Pseudomonas aeruginosa* (4 cases, 2.3%). In sum, 93 cases (54.1%) were caused by Gram positive bacteria, 12 (7.0%) were Gram negative bacteria, 2 (1.7%) were Mycobacteria, and 6 (3.5%) were yeast infection. Fifty-nine cases (34.3%) were culture-negative. The result is listed in Table 2. There was no significant difference between the two groups.

**Table 2:** Culture result of patients with or without chronic hepatitis

	Non-hepatitis	Hepatitis
*Staphylococcus aureus*		
OSSA	39 (26.5%)	5 (20%)
ORSA	5 (3.4%)	0
Coagulase negative *Staph.*	27 (18.4%)	5 (20%)
Group B *Streptococcus*	3 (2.0%)	0
*Enterococcus faecalis*	5 (3.4%)	1 (4%)
*Escherichia coli*	2 (1.4%)	0
*Pseudomonas aeruginosa*	4 (2.7%)	0
*Morganella morganii*	0	1 (4%)
*Parvimonas micra*	0	1 (4%)
*Serratia marcescens*	2 (1.4%)	1 (4%)
*Pseudomonas stutzeri*	1 (0.7%)	0
*Mycobacterium* spp.	2 (1.4%)	0
Yeast spp.	4 (2.7%)	2 (8%)
Culture negative	53 (36.1%)	9 (36%)

## Discussion

With the two-stage exchange arthroplasty treatment protocol, a significantly higher incidence of infection recurrence was noted in the hepatitis group compared to the non-hepatitis group (28% vs. 9.5%) in this study. For the hepatitis group, whether the pathogen is HBV or HCV does not affect the outcome.

It has been well known that chronic liver disease is associated with higher surgical mortality and morbidity rates. HBV can suppress the production of primary cytokines involved in the innate immune response, disturbance in proliferation processes, and cytokine production [22]. Chronic infection with HCV often develops into liver cirrhosis [28, 29]. Therefore, numerous studies have revealed that chronic liver disease is associated with higher surgical mortality and morbidity rates. In non-orthopedic operation, studies have reported a perioperative mortality rate up to 25% [30–32]. Furthermore, complications such as bleeding, infection, and multi-system failure have also increased in patients with chronic liver disease [33].

In this study, all seven patients with chronic hepatitis who failed two-stage exchange arthroplasty underwent prosthesis removal and a second two-stage exchange arthroplasty. The patients were followed for at least 21 months in the outpatient department (mean, 37 months; range 21-68 months), and no sign of recurrent PJI was noticed.

Liver cirrhosis has been recognized as a risk factor for hip PJI treatment failure with two-stage exchange arthroplasty [34, 35]. In this study, we further divided knee PJI patients with chronic hepatitis into with or without liver cirrhosis. Demographics of patients are listed in Table 3. Although not significant, the infection recurrence rate was higher in the cirrhosis group than the non-cirrhosis group (100% vs. 21.7%). Further investigations may be required to determine whether knee PJI patients with liver cirrhosis should consider other treatment protocols such as fusion or amputation.

**Table 3:** Demographics and characteristics of the hepatitis patients with or without liver cirrhosis

	Non-cirrhosis	Cirrhosis	*P*
Number	23	2	
Age	63 (30-83)	72 (68-76)	0.280
Sex			0.480
Male	12 (52.2%)	0	
Female	11 (47.8%)	2 (100%)	
F/U months	39 (13-85)	36 (28-43)	0.837
Infection relapse PJI	5/23 (21.7%)	2/2 (100%)	0.070**
Charlson comorbidity index	5 (1-7)	6 (6-6)	0.353
Treatment after failure			
Second revision	5	2	
DAIR	0	0	
DAIR, failure, 2nd revision	0	0	
Debridement	0	0	

According to the Delphi-based International Consensus of PJI 2013, DAIR can be performed on PJI patients who had late hematogenous infection within 3 weeks of an inciting event or symptoms no longer than 3 weeks [36]. In the non-hepatitis group, 4 patients were treated with DAIR, with a success rate of 75%. Several risk factors have been identified as early predictors of treatment failure in the DAIR procedure. Tornero et al. established the Kidney, Liver, Index surgery, Cemented prosthesis and C-reactive protein value (KLIC) score in a retrospective study of 222 procedures [37]. They found 5 independent pre-operative predictors of failure, including chronic kidney disease, liver cirrhosis, infection of a revision arthroplasty or arthroplasty for femoral neck fracture, cemented prosthesis, and presenting C-reactive protein > 11.5 mg/dL. If the KLIC score is greater than 6, DAIR is more likely to fail. Whether DAIR is suitable for patients with chronic viral hepatitis needs more larger-scale and prospective studies in the future.

There are several limitations in this study, including minimum length of one-year follow-up, small sample size, and retrospective study design. Larger prospective randomized controlled trials are needed to confirm whether the selection criteria for two-stage exchange arthroplasty after the first episode of PJI can improve prognosis and quality of life in patients with chronic hepatitis. This study suggests that surgeons can consider two-stage exchange arthroplasty when counseling knee PJI patients with chronic hepatitis, and provides a basis for future studies.

## Conclusion

Knee PJI patients with chronic hepatitis displayed a higher rate of treatment failure (28%) after two-stage exchange arthroplasty and needed further surgery for infection control.

## Data Availability

The datasets used and analyzed during the current study are available from the corresponding author on reasonable request.
